# Supplementary material for: A Novel Nutrient- and Antioxidant-Based Formulation Can Sustain Tomato Production under Full Watering and Drought Stress in Saline Soil
Source: Plants (Basel). 2023 Sep 27;12(19):3407. doi: 10.3390/plants12193407 (PMC10574430; doi:10.3390/plants12193407)
Supplement: Supplementary file 1 [file plants-12-03407-s001.zip › plants-2575353-supplementary.pdf]

Table S1. Primers sequences for RT-PCR of the stress-related genes in tomato.

The gene	Reference Seq.	5′ - 3′ primer sequence	T_A
Actin 1	*AB181991*	F: CTCTGACAATTTCCCGCTCA<br>R: ACACGCTTCCTCATGCTATCC	58 °C
Actin 2	*AT2G37620*	F: GCTATTCAAGCCGTGCTTTC<br>R: AGCATGTGGAAGGGCATAAC	
SOD	*MG893090.1*	F: TTCGCCATGCTGGTGATCTT<br>R: CATGGACAACTACGGCCCTT	
CAT	*GU984379*	F: GGCTGCTTGAAGTTGTTCTCCT<br>R: CTGCTAGTACCTCCTGATCCGTT	
APX	*KU747079.1*	F: TGGCCTGCTCTTCCTCTAGT<br>R: CATGCCACGCTAATCGAAGC	
GR	*KX828561.1*	F: CAACGCGCTTTGGTAACTCC<br>R: GGGCCCTAATGAAGTGGAGG	
